# Once the Internet can measure itself

**DOI:** 10.1098/rsta.2014.0437

**Published:** 2016-03-06

**Authors:** Scott Kirkpatrick

**Affiliations:** School of Engineering and Computer Science, Hebrew University, Jerusalem 91904, Israel

**Keywords:** Internet measurement, monitoring, complex systems, graph topology

## Abstract

In communications, the obstacle to high bandwidth and reliable transmission is usually the interconnections, not the links. Nowhere is this more evident than on the Internet, where broadband connections to homes, offices and now mobile smart phones are a frequent source of frustration, and the interconnections between the roughly 50 000 subnetworks (autonomous systems or ASes) from which it is formed, even more so. The structure of the AS graph that is formed by these interconnections is unspecified, undocumented and only guessed-at through measurement, but it shows surprising efficiencies. Under recent pressures for network neutrality and openness or ‘transparency’, operators, several classes of users and regulatory bodies have a good chance of realizing these efficiencies, but they need improved measurement technology to manage this under continued growth. A long-standing vision, an Internet that measures itself, in which every intelligent port takes a part in monitoring, can make this possible and may now be within reach.

## 1. Introduction

At this discussion meeting, it has been exciting to hear that data already circle the UK at 3 terabits per second, with even greater bandwidths possible through multicore cable, multimode fibre and similar parallel transport approaches. But the weak link in chains of communication has always been the interconnections. In this paper, we consider not the electronic connections between lengths of fibre but, in the Internet, the points at which the 50 000 or so subnetworks (called autonomous systems, or ASes) connect to each other. Each of these subnetworks is under the control of a different organization, which may be a data carrier, a content provider, a service provider, a government office, a company or even a university. Each of these is a business with its own unique objectives, and the interconnections at the points where ASes meet require business contracts. When problems arise that cannot be quickly solved, such as prime-time congestion at heavily loaded connection points, they can only be resolved through negotiation which arrives at new contractual terms. Naturally, this is not a quick process; detecting and determining the cause of any problem takes time as well, ranging from minutes to months.

In this paper, we focus on how the topology of the Internet, viewed as a graph of all the connections between ASes, can affect its overall performance. Knowing the Internet topology is critical to understanding throughput and capacity of the Internet under its heterogeneous workload. This is hard to know because it is not specified in any blueprint, not even documented in any single place or very accurately. The graph of AS connections just grows in response to market forces and business decisions, many of which are treated as sensitive and proprietary information. But several social and legal forces are coming to bear on the Internet’s providers, making it possible to see more clearly how the network operates, and turn that knowledge to public advantage.

## 2. Net neutrality and transparency

Recently in the USA and in Europe, governments and their regulators have given attention to the question of how best to manage this complexity and reduce its uncertainty for the benefit of end users in homes and offices, companies of all sizes, all without unduly impacting the profitability of the data-carrying operators who make it possible. At a recent workshop,^1^ Scott Jordan, the chief technical advisor to the Federal Communications Commission (FCC; the US communications regulator) and Frode Sorensen of BEREC (the European board of electronic communications regulators) were asked to compare the approaches being taken in the two domains. A quick summary of their two positions, both similarities and differences, follows.

Network neutrality has a similar meaning on both sides of the Atlantic, but there are significant differences in the details of its implementation. The FCC has established clear principles: there shall be no blocking, throttling or ‘paid prioritization’ (also termed ‘fast lanes’) distinguishing one class of packets from another on the basis of their contents or apparent purpose.^2^ But implementation of these principles in the USA has not started, litigation looms, and in which parts of the Internet (initially, close to the edge) neutrality will be required is a subject under continuing review. ‘Normal network management practices’ will be allowed some exceptions, as long as they do not discriminate against any class of users or uses. Clarifying just what network management practices are normal and non-discriminatory will require adjudication of actual cases in which complaints arise.

In Europe, three countries already have net neutrality regulations, starting with Norway.^3^ Bandwidth caps which discriminate between different types of services are illegal in Norway, but allowed in The Netherlands. ‘Special services’, such as Virtual Private Networks (VPNs), are approved in Europe as long as they are isolated from end to end from the standard Internet access offerings. Since both standard and special service data traffic are generally multiplexed across the same equipment, it is not at all clear how this isolation can be convincingly shown. Blocking or throttling (slowing) packets of certain descriptions (e.g. Voice over Internet Protocol (VoIP) or peer-to-peer file sharing) is observed to occur in as many as one-third of European subnetworks.

Examples in which network management has meant under-provisioning a critical interface as part of a business negotiation between a content provider (e.g. Netflix or YouTube) and an Internet Service Provider (ISP) providing the only path to a great many homes are easy to find [1,2]. They have the clear side effect of making it very hard to get bandwidth for other, unrelated Internet activity from or to these homes. These situations do not appear to violate net neutrality or fairness in a legal sense, as all traffic, not just Netflix, feels the blockage. So we need to appeal to the as-yet ill-specified notion of ‘transparency’ if we wish to see them exposed to public scrutiny, and resolved more quickly because of this pressure. The traditional view of Internet connectivity is that end users have many choices from which to select their access provider ISP and that content providers have many points through which they can direct their content to the end user. But this is clearly not the case in the USA, where two ISPs, Verizon and Comcast, are the access providers for the majority of home customers. In fact, our view of the structure of the Internet today has come a long way from the traditional picture, taught in textbooks.

## 3. How is the Internet interconnected?

In the original, hierarchical picture of the Internet’s structure, carriers are layered in tiers. A message from a customer at the edge to a distant destination is passed through its access provider, a Tier 3 participant, to a higher-level Tier 2 carrier. If the destination is served by the Tier 2 carrier or its Tier 3 underlings, it delivers it. If it is further away, it passes the message to a Tier 1 carrier, one with global extent to its services. The Tier 1 carrier finds a Tier 2 carrier which serves the destination region, and that carrier sends the message to its destination via a Tier 3 carrier which serves the user or enterprise at the other end. Each time a message is handed up or down in the hierarchy, a small charge is collected. But peering, in which, for example, a Tier 2 carrier passes a message to another Tier 2 carrier who has as a customer the Tier 3 carrier to which the message is directed, also serves the interests of the two carriers at the same level, since each is being compensated by a customer at one or the other end of the message. Therefore, no extra charge might be required for a peering interchange.

While the purely hierarchical structure makes determining a route for a message simple, and each participant is being compensated for their efforts, it is fragile and quickly becomes congested. A measure from graph theory, called betweenness,^4^ captures this. Assume a simple traffic model: every customer sends one message to every other. Betweenness for a carrier is the number of messages that they must transport in this traffic model. In the naive hierarchical picture of the network, there are only half a dozen Tier 1 carriers, a few hundred to a thousand Tier 2 carriers, and 20 000 or more Tier 3 or access (‘eyeball’) ISPs. As a result, nearly all of these messages must pass through one of the Tier 1 carriers. With a billion or more people using the Internet, the 10^18^ messages that are produced, concentrated at the handful of Tier 1 carriers, would overwhelm even a multi-terabit backbone.

As a result, peering between regional carriers at each level has developed as a common means of offloading traffic from the Tier 1 carriers (and saving on the transit charges that would have been incurred). This ‘flattening’ of the Internet has been widely noted. Because it is the result of many private and proprietary agreements in which mid-tier carriers agree to accept traffic not only for their own customers, but for transit, simply determining and monitoring the structure of the AS graph of the Internet has become an interesting and active problem. In addition, new entrants to the Internet community have appeared whose business is providing content, such as videos, online catalogues and the retail services behind them, or simply transporting such data for other enterprises to multiple caches closer to the ultimate customers. Content distribution operates under a business model quite different from pure transport. Thus, one would expect that the arrangements that content distribution networks (CDNs) and content providers (CPs) make would be different from the deals which accompany interconnections between local, regional and global data transport operators, yet little is known about what form such arrangements take.

As part of the Border Gateway Protocol (BGP) by which ASes determine how to route traffic between them, each border router broadcasts long lists of the routes that it will serve to various destinations. Collecting these broadcasts and extracting the AS–AS links between them is one way of monitoring the current state of the AS graph, but it tends to present only the (paid) transit links. To observe peering links, several groups (e.g. CAIDA,^5^ RIPE^6^ and DIMES^7^ [4]) have used tools such as Traceroute [5], which sends a stream of packets to a single destination from a single observation point, with each successive packet reporting the address it has reached after an increasing number of hops. As long as the packets all follow the same path, this traces the actual links along which the message has been routed, and peering links are in fact observed. The effectiveness of Traceroute observations is difficult to quantify (how to count the links not seen?), but it clearly depends both on the completeness of the set of destinations to which traces are sent and on the richness and coverage achieved by the observation points from which they are sent. Solutions to the first problem have been worked out by groups from CAIDA, DIMES and RIPE in the past, but establishing and maintaining enough points from which to see deep into the Internet remains the bigger problem, which we will discuss below. A curious difference between Traceroute-based discovery of the links in the Internet and identifying links by collecting the routes advertised by BGP routers is that the two methods appear to discover different sets of links. In the DIMES studies, Traceroute from several thousand downloaded software clients discovered fewer ASes and fewer links overall than the BGP data collected by the RouteViews project.^8^ It added only a few hundred ASes not seen already by RouteViews, but the links discovered by DIMES increased the total number of links seen (in either direction) by about 30%. When a link was discovered by both methods, it was frequently found that the two methods reported passing the link in opposite directions. This suggests that the Traceroute data are reporting links found during the ‘upward’ path from the edge of the Internet to its centre, while BGP emphasizes the ‘downward’ paths which their ASes offer to eventual destinations, also suppressing peering links that are offered on a more limited basis. This hypothesis is consistent with subsequent work in which Traceroute studies conducted from a few large Internet Exchange Points (IXPs) and ISPs do discover some peering routes, but add only a per cent or so of total links ([6] and references therein).

If we have sufficient data about the AS graph, we can try to learn more about the roles of different participants from looking at their connectivity—not just their degree, *z* (the number of connections to other ASes), but the flexibility with which a particular AS can connect with the rest of the Internet. An elegant decomposition of the graph into its ‘*k*-shells’ [7,8] accomplishes this, where *k* is the number of independent ways in which a given AS connects to the rest of the Internet. If we prune from the graph all nodes (ASes) which have *k* or fewer neighbours and keep doing this until all the nodes remaining have more than *k* connections to the other nodes remaining, the decomposition is unique and the pruning search can be programmed to run in time which is nearly linear in the number of nodes. Suppose we do this in successive stages, starting with *k*=1, increasing *k* by 1 at each stage. The ASes which we remove at the *k*th stage make up the *k*-shell. Ordering the ASes by the *k*-shell in which they participate is more nuanced than labelling them as members of Tiers 1, 2 or 3, yet seems to capture the role of the higher layers in carrying messages further and more flexibly without presuming to know anything about the business relationships involved. If we lump together and analyse the nodes in shells pruned with values of *k* or less, this ‘*k*-crust’ captures the capabilities of the subnetworks which make up the edges of the Internet. The nodes remaining with more than *k* interconnections constitute a *k*-core. As *k* becomes large, these are the ASes which constitute the backbone of the modern, post-textbook Internet.

**Figure 1.**
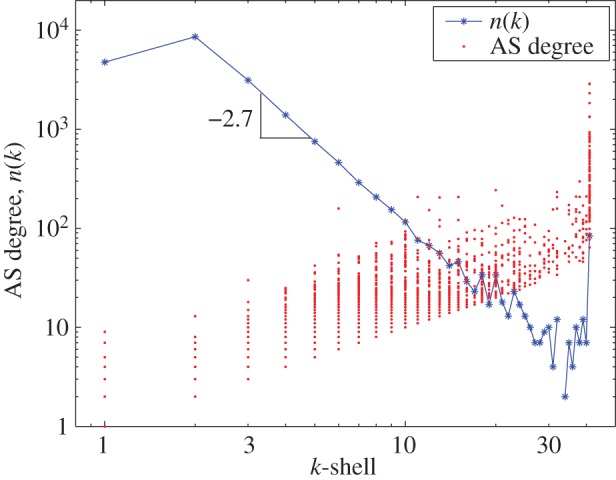
Number of ASes, *n*(*k*), in each *k*-shell, which decrease in proportion to *n*^−2.7^, and scatter plot of the degrees, *z*(*k*), of those ASes, plotted against *k* (adapted from [7]).

We plot in figure 1 the number of ASes found in each *k*-shell (using some rather old data, but the same pattern has been found in subsequent years), and at each value of *k*, provide a scatter plot of the initial degree, *z*, of the ASes in that shell. The population of the *k*-shells (the blue line in figure 1) decreases rapidly, as a power law in fact. The pruning process stops at a final value of *k*, not with a handful of recognized global ‘Tier 1’ carriers, but with a dense nucleus of about 100 ASes, each pair of which connect to each other over 50–100 distinct routes. Over 70% of the pairs of ASes in the nucleus have direct links. With regard to the betweenness catastrophe sketched earlier, the nucleus of the Internet functions as a giant non-blocking crosspoint switch, reducing the number of messages that must traverse any single link in the Internet by the square of the number of participants, or as much as 10 000 times.

**Figure 2.**
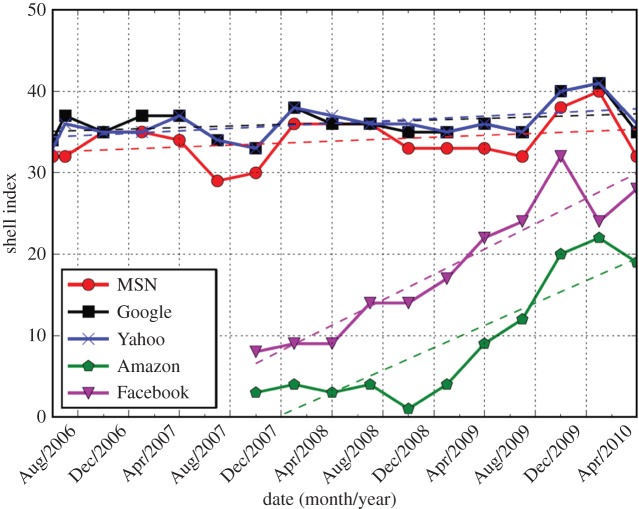
The *k*-shell index of five major content providers (adapted from [8]).

The ASes in the nucleus have achieved this level of interconnection for several different business purposes—they are CPs and CDNs as well as transit providers of the more traditional sort but coming from many large countries as well as from the USA. The scatter plots in figure 1 show that the degrees, *z*, of the nucleus ASes range continuously over about a factor of 100, with the classic intercontinental transit carriers having the largest values of *z* and the CPs dedicated to distributing their own content the smallest. This contrasts with the degree variation of about a factor of 10 seen in the earlier shells. Recent topology data from the DIMES project [9], shown in figure 2, track the progress of the larger CPs during the period 2005–2010. We see that Google, Yahoo and MSN have been nucleus members throughout the period, while Amazon, which earlier had relied on CDNs such as Akamai to distribute their content, and Facebook, a newer entrant, appear at shells with steadily increasing values of *k*, and by today have joined the core group.

A number of researchers have pointed out what is termed the ‘flattening’ of the Internet.^9^ Some evidence comes from traffic studies which inspect the actual sources and destinations of flows sampled well within the Internet. These show that in recent years much of the traffic has come to be the result of distributing multiple copies of popular content to cacheing sites around the world. And it appears to flow to its destinations without involving the traditional Tier 1 carriers. This is supported by a second reason to expect flattening—that as the middle-level carriers develop private peering arrangements with each other, the need to involve the expensive global transit carriers becomes less.

**Figure 3.**
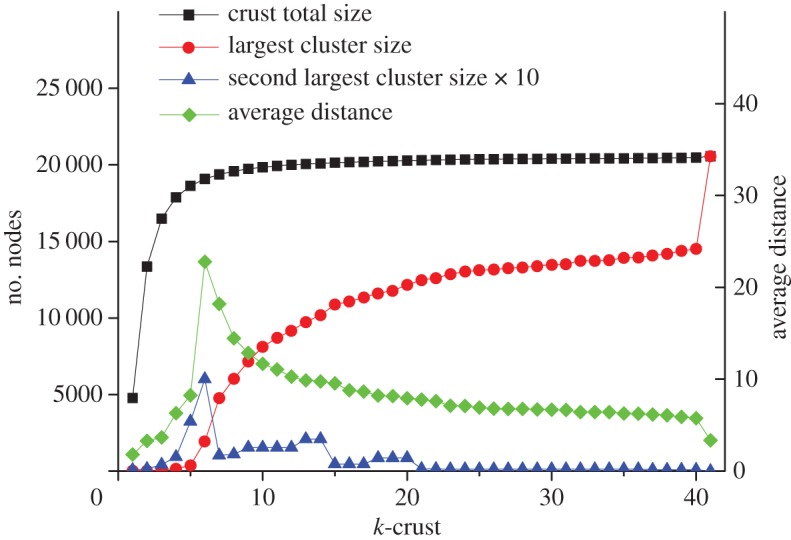
Characteristics of the *k*-crusts (adapted from [6]).

There is a simple way to test for this possibility with the AS graph information, using the structure of the *k*-crusts. In figure 3, we show a number of interesting statistics of the *k*-crusts, plotted against *k*. As a test of the effectiveness of peering to complete data transport, we plot the size of the largest cluster found within the *k*-crust and the average distance between pairs of sites for each value of *k*. For *k*<5, the crusts consist only of small isolated groups of ASes, but for *k*>5 a single large connected cluster forms, and the average distance between pairs of ASes (number of interfaces that must be traversed) rapidly drops. The potential for long-range transport using only peer connections clearly exists, but it requires more AS to AS interconnections, and may not be preferred under the standard business arrangements. One intriguing feature of the structure shown in figure 3 is that roughly one-third of the ASes do not participate in this highly connected crust, but make their connections directly and solely into the nucleus or core of the Internet. The data plotted in figure 3 were gathered in 2005, so an effort is currently under way to revisit this analysis with data for the 2015 AS topology. Further ‘flattening’ of the Internet should increase the size of the nucleus, and perhaps accelerate the sharpness of the growth of the connected component of the crust and decrease the fraction of ASes that are dependent on the nucleus carriers. But this remains to be seen, and it will be interesting to see if this structure can be understood through some of the evolutionary modelling efforts which are currently being developed to treat the game theory aspects of the AS ecosystem. At any rate, it is clear that a more realistic traffic model than the ‘betweenness’ calculation has to be developed to more accurately estimate the capacity limits within the Internet as a whole and in its core in particular.

## 4. The role of measurement: and new opportunities

Stakeholders need to know more about this topology and how it is evolving. Who are these stakeholders? End users are one group, because a choice of Internet access supplier can affect the quality of the experience they derive by making it easier or more difficult for them to reach the services they most need. National regulators have recently become concerned with assuring that advertised bandwidths and reliability of Internet access are actually delivered to customers in their countries. Assuring a ‘level playing field’ for services and enterprises in their jurisdictions is a next step. Small-to-medium-size enterprises throughout the world, those not big enough yet to be playing with the ‘big dog’ CPs in the nucleus of the Internet, need to know the implications of their choices in delivering their services to customers in their own regions and ultimately around the world. The measurements needed to keep close track of the topology have been difficult in the past, the province of a few research teams, but this may be about to change.

Several non-profit consortia and companies monitor the performance of the Internet from its edges at broadband^10^ modems and at cellphones.^11^ With the support of national regulatory agencies in the USA and in Europe, companies like SamKnows have placed in excess of 40 000 active measurement boxes (wireless modems, reprogrammed over a standard Unix operating system subset) as close as possible to the DSL or cable entry point, in order to measure the quality and reliability of broadband access at homes. One company that I have worked with, WeFi,^12^ is monitoring Internet characteristics from smart phones, tagging its measurements with the application currently or most recently running on the phone, and logging information characterizing that application’s performance. This gives much information about human activity, and its Internet information is centred on the things that end users are most interested in doing. Operating on a smart phone offers some of the advantages of passive measurement, because one can record actual application performance, as seen inside the phone, in addition to simulating standard actions such as up- and downloading files, as is done in most active measurement. They report that their application is now running on over a million smart phones, mostly in the USA but also throughout the rest of the world.

**Figure 4.**
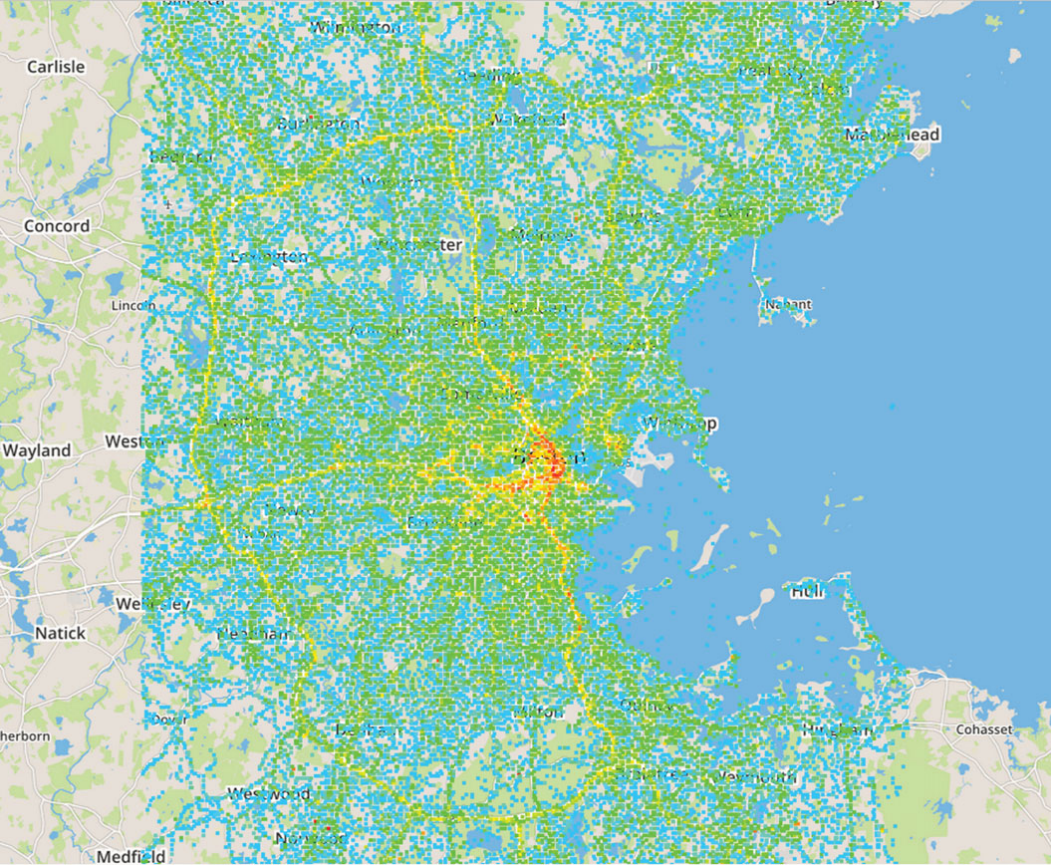
Spatial distribution of WeFi measurements in the Boston area, November 2014. Theseclients had a mapping application active and were using a cellular carrier to access the Internet.

Many times more smart phones are sold every year than laptops or computers, so the number of observation points that can be created by putting a monitoring application on smart phones is potentially much larger than has been available in the past. In the future, such small mobile devices may become the true edge of the Internet. In figure 4, we show the map of the frequency with which measurements are obtained by WeFi in the Boston area, integrated over the month of November 2014. About 16 000 different phones contributed measurements during the course of the month. Roughly, 7000 were seen on any single day. We have somewhat narrowed the associated human activities by filtering for the map only those phones that were running a mapping application and using the cellular carrier for their Internet connection. That tends to favour drivers in their cars, getting navigational and traffic information. Data are logged more often when the phone is moving, so of course the highlights on the map are the major highways in the Boston area. In the Los Angeles area, for the same time period, we acquired even more data, sampling from the northwestern third of the city: 50 000 phones, of which about 20 000 were observed each day.

**Figure 5.**
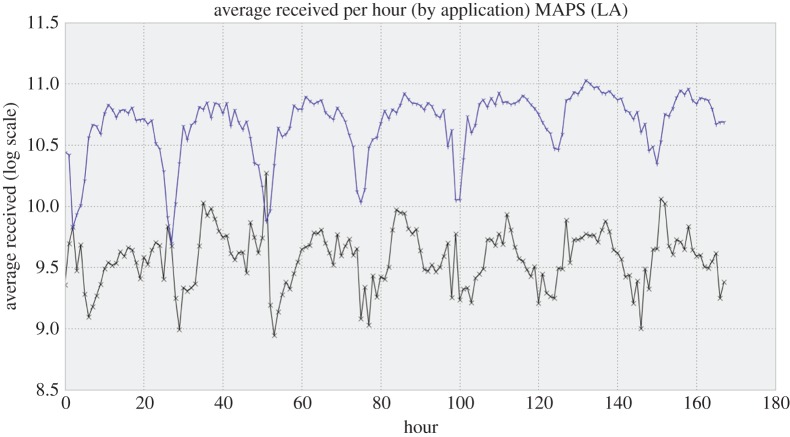
Diurnal variation of data download performance experienced by WeFi clients while running one of two mapping applications in the Los Angeles 4 week dataset. The vertical axis is the average received bandwidth; the horizontal axis is the time of the measurement expressed in hours since the previous Friday midnight. The upper curve depicts the performance of Google Maps, and the lower curve is that of WAZE.

Further filtering shows that the phones produce interesting data 24 h a day, 7 days a week, and looking for diurnal variation (how the average performance of things varies with the hour of the day or week) provides a useful way of seeing what clues this flood of data can provide about the Internet’s overall quality of service and the resulting quality of user experience. For example, in figure 5, we show the variation over the course of a week (averaged over four weeks, in fact) of the download performance achieved by two mapping applications, Google Maps and WAZE’s traffic and navigation application (which was in fact acquired by Google before these data were taken). We notice that the daily pattern is different between these two similar applications. One possible reason for the different patterns was discovered when I ran Traceroutes to the two applications from my own smart phone, while sitting at the Los Angeles airport (LAX), and found that the servers for the two applications are located in two different cities. Map updates must reach the phones by different routes, and thus may experience different network performance.

This sort of Internet ‘health monitoring’, taking advantage of the highly dispersed population of cellphones, can provide an early warning of congestion problems that occur at the interfaces between the ASes of the steadily evolving Internet. Can the smart phone monitors also help in tracing changes in this topology? With more advanced measurement tools, more like the probes used by research groups such as CAIDA and RIPE, I do not see why this huge population of potential monitors, aided by all the ‘big data’ tricks that are now available to manage the flood of data that will result, cannot give us this information. Expert hands may still be required to bore down and determine the root causes of Internet failures or congestion problems (as they have in several recent cases). But an Internet that monitors itself, identifies issues and recommends means of relief, may be within our grasp, with the capability to provide true transparency, a clear view into the Internet, representing the interests of its consumers. The discussion of how the goals of transparency and network neutrality (or a ‘level playing field’) can be combined to achieve this while balancing national, consumer and business interests, will be interesting.

## Footnotes

1 See http://internet-monitoring-study.eu/index.php/workshops, contains material presented to the study and reports summarizing the workshops.

2 See https://www.fcc.gov/document/fcc-releases-open-internet-order.

3 Sorensen’s views are summed up in a series of articles collected at http://eng.nkom.no/technical/internet/net-neutrality/on-the-origin-of-specialised-services.

4 See http://en.wikipedia.org/wiki/Betweenness_centrality for an overview. For the best algorithm with which to compute it, see [3].

5 See www.caida.org.

6 See www.ripe.net.

7 See www.netdimes.org.

8 See www.routeviews.org.

9 A recent paper which reviews the ‘flattening’ literature is [10].

10 See https://www.fcc.gov/measuring-broadband-america; https://www.samknows.com; www.leone-project.eu.

11 See https://www.monroe-project.eu/.

12 See www.wefi.com.
